# Evaluation of fitness parameters in relation to racing results in 245 Standardbred trotter horses submitted for poor performance examination: A retrospective study

**DOI:** 10.1371/journal.pone.0293202

**Published:** 2023-10-19

**Authors:** Chiara Maria Lo Feudo, Luca Stucchi, Giovanni Stancari, Bianca Conturba, Chiara Bozzola, Enrica Zucca, Francesco Ferrucci

**Affiliations:** 1 Equine Sports Medicine Laboratory “Franco Tradati”, Department of Veterinary Medicine and Animal Sciences, Università degli Studi di Milano, Lodi, Italy; 2 Department of Veterinary Medicine and Animal Sciences, Veterinary Teaching Hospital, Università degli Studi di Milano, Lodi, Italy; University of Montenegro, MONTENEGRO

## Abstract

In racehorses, the associations between physiological parameters obtained by exercise testing and racing results have been questioned. We hypothesized that fitness variables measured during a treadmill incremental test may be related with racing outcomes and lifetime career. Our study aimed to investigate the role of fitness parameters obtained during a treadmill test in performance evaluation and career prediction in poorly performing Standardbreds, through a retrospective review of the clinical records of 245 trotters that underwent an incremental treadmill test. Several fitness parameters were assessed, and their correlations with racing data (number of starts, wins and placings) in the 3 months before and 6 months after examination were evaluated; moreover their associations and predictive power for lifetime racing results and earnings were considered. The associations between fitness parameters and measures of racing performance as well as the associations between racing results over time were evaluated by Spearman’s correlation in the whole population and in different age groups. Multivariate regression models predicting the total number of starts, wins and earnings based on fitness parameters were constructed, controlling for age, weight, and sex. Maximum speed and the speed at the aerobic threshold were the parameters correlated with the most racing results, in the three evaluation periods (3 months before, 6 months after, lifetime). Other parameters predictive of career outcomes were maximum blood lactate concentration and maximum hematocrit. Interestingly, in 2-year-old Standardbreds, the only parameters correlated with racing results were maximum hematocrit and minimum pH, suggesting they may reflect individual potential. Both the racing results obtained before and after examination were predictive of lifetime career, with those following hospitalization being more strongly correlated. In conclusion, parameters obtained during treadmill tests both reflect the racing outcomes and the career potential.

## Introduction

Poor athletic performance is a common issue in racehorses, usually related to paraphysiological or pathological limitations to energy production [1]. Throughout the decades, researchers have widely discussed the most appropriate approach for the objective quantification of changes in performance decrease, without reaching univocal conclusions. Studies on poor performance either rely on trainers’ and owners’ opinions [2–4], reflecting their subjective impressions and expectations, or attempt to profile and quantify performance through measurable parameters. In particular, the assessment of various fitness parameters, obtained during and/or after exercise tests, has been proposed [5–7]. Moreover, different racing outcome measures have been adopted in previous studies, including number of starts, wins, placings, earnings, racing times, and statistically modelled measures of performance (i.e., speed figure, a function of racing time adjusted for racing conditions) [8–11].

With the aim to identify measurable fitness parameters capable of predicting racing results, many studies investigated the correlation between blood lactate concentration, heart rate and racing performance. Significant correlations between the speed at which the blood lactate concentration reaches 4 mmol/L (VLa4), measured during exercise tests on treadmill or on track, and racing times [12–14], earnings [13], index of trot (the official French annual index of performance) [13], and placings [5] have been reported by several authors in Standardbred trotter horses of different performance levels; conversely, in another study, the associations between VLa4 and performance indices were weak in well-performing pacers [15]. Moreover, the relationship between racing performance and post-exercise lactate concentrations is debated [1, 16, 17].

Other investigated fitness parameters include the heart rate during different steps of an incremental exercise test, which seems to be correlated with racing positioning in a population of well- and poorly-performing Standardbreds [5], and the heart rate at the end of a treadmill exercise test, which was associated with handicap ratings in fit Thoroughbreds [17]. The speed at which a heart rate of 200 bpm is reached (V200) was associated with index of trot, earnings [13], placings [5] and racing times [13] in two studies on Standardbreds of different performance levels, while other authors found no relationship with the best racing time in fit Standardbreds [14]. Interestingly, a correlation between hematocrit increase after splenic contraction and racing times and placings was reported by two studies [5, 18], while another one failed to detect any significant association [17].

The reasons for these discrepancies in the results obtained by different research groups are probably attributable to the characteristics of population included (good performers/poor performers/mix of good and poor performers), the type of exercise test (on treadmill/on field, incremental/submaximal/maximal, etc.), and the racing outcomes chosen for comparisons. The need for a consensus on which measures properly quantify racing performance has recently been highlighted, as some of them only reflect the athletic longevity of racehorses, and others the quality and success of their career [19]. Moreover, as physiological responses during exercise can be efficiently improved by training [20], it is possible that individual variations become less obvious with increasing age and training status.

We hypothesized that fitness parameters measured through exercise tests may be predictive of the racing career in poorly performing racehorses, and that their relevance may vary with age. Moreover, as diagnostic procedures reveal the horses’ condition at the time of examination, we also hypothesized that the obtained fitness parameters may be associated with racing results in limited periods surrounding the time of examination. Therefore, the present study aimed to compare a range of fitness parameters obtained during a standardized treadmill test with various racing outcome measures, divided per periods of time. Furthermore, the possible value of fitness parameters and racing outcomes, at the time of examination, in the prediction of the lifetime racing career of Standardbred trotter horses was investigated.

## Materials and methods

### Ethics statement

Ethical review and approval were not required for this study, as it consists of a retrospective study on clinical equine patients. All the procedures were performed for diagnostic purposes according to the Code of Good Veterinary Practice and relevant guidelines, as a part of standard diagnostic protocols; no procedures were performed for research purposes. Written informed consent for the use of clinical data for research was obtained from the animals’ owners or holders.

### Study population

A retrospective study was performed by reviewing the medical records of the Standardbred racehorses that underwent an incremental treadmill test at the Equine Sports Medicine Unit (Veterinary Teaching Hospital, University of Milan, Italy) between 2002 and 2021, and comparing them to the racing results of each horse. The study population consisted of 245 horses that were in full training upon admission and referred to our Hospital for poor performance examination. Besides physical examination, blood laboratory analysis, baseline electrocardiogram (ECG), and incremental exercise test on the treadmill, which were performed in all horses, further diagnostic procedures were selected based on history, clinical findings, and diagnostic suspicion.

### Incremental treadmill test

Horses were first conditioned to the use of the high-speed treadmill (Sato I, Uppsala, Sweden) by one or two training sessions; then, an incremental treadmill test was performed according to a standardized exercise protocol, as follows. Horses were tacked with the racing equipment and wore a heart rate meter (Polar, Equine Inzone FT1, Steinhausen, Switzerland); to allow blood collection during the test, a venous catheter was placed in the left jugular vein and connected to an extension tube. The belt was inclined with a 5% slope. After a warm-up phase of 4 min walking (1.5 m/s) and 3 min trotting (6 m/s), the protocol started, consisting of 1 min phases of increasing the speed by 1 m/s, until the onset of fatigue, when the horse was no longer able to maintain the treadmill speed. Then, horses were walked with 0% slope for 30 min to cool down [21]. At the end of the warm-up, at the end of each speed phase, and at 1, 5, 15, and 30 min during the cool-down, blood was collected. Samples of 1 mL were transferred into tubes with 10 mg sodium fluoride and 2 mg potassium oxalate, centrifugated and refrigerated. The enzymatic colorimetric method was used to assess plasma lactate concentrations, through a lactate dry-fast kit for the automatic system (Uni-Fast System II Analyzer; Sclavo Diagnostics) and its own reagents. Moreover, according to the availability of the required analyzer and equipment during the years, an aliquot of blood at each phase was transferred into heparinized syringes and blood pH (*n* = 162) and hematocrit (*n* = 121) were measured by a blood gas analyzer (IL 1630, Instrumental Laboratory; CCA blood gas and electrolyte analyzer, Opti Medical Systems) [22]. Among the fitness parameters obtained during the incremental test on treadmill, the speed at a heart rate of 200 bpm (V200) and the speed at the aerobic-anaerobic threshold, corresponding to a plasma lactate concentration of 4 mmol/L (VLa4), were selected as good indicators of the aerobic capacity of horses [5, 23, 24]; the exact values of VLa4 were calculated through a specific software, providing precise lactate threshold markers by inverse prediction (Lactate-E 1.0, David Higgins) [25]. Moreover, the peak plasma lactate concentration (Lac max), index of both the aerobic and anaerobic capacity [17, 26], the minimum pH (pH min), reflecting blood acidosis and buffer capacity, and the maximum hematocrit (Ht max), reflecting oxygen carrying capacity [27], were considered. Finally, the maximum speed reached until the onset of fatigue (V max) was registered.

### Race data collection

Race data were extracted from the public official online database for Italian horseracing (HiD Ippica, www.hippoweb.it, Italy). Collected data included lifetime starts, wins, placings (top 3 finish), and earnings as well as the number of starts, wins, and placings during the 3 months before and the 6 months after examination. From obtained data, the ratios between number of wins and number of starts (W:S), number of placings and number of starts (P:S), and earnings and number of starts (E:S) were calculated. The period of the 3 months before admission was chosen to represent the period of poor performance, while the following 6 months represented the post-treatment period. At hospital discharge, the veterinary team prescribed treatment based on the identified disorder, although treatment was not confirmed and no follow-up examinations were performed. Lifetime racing results were considered only for horses that, at the moment of the study, have retired from racing (*n* = 238).

### Statistical analysis

Data was collected on an electronic sheet and analyzed using two commercial statistical softwares (GraphPad Prism 9.5.0; Stata 17). Normality was evaluated by Kolmogorov-Smirnov test with Dallal-Wilkinson-Lillie *p* value, and descriptive statistics were performed.

A correlation matrix was used to evaluate the associations between fitness parameters and race results. To evaluate the effect of age on these correlations, correlation matrices were also designed for each age group of horses based on classical racing categories as follows: 2-year-old, 3-year-old, ≥ 4-year-old. The associations of race results before and after examination with the lifetime career outcomes were investigated by Spearman’s correlation. Statistical significance was set at *p* < 0.05. Data was summarized with mean ± standard deviation (SD) if normally distributed, or with median and interquartile range (IQR) if not normally distributed.

For the prediction of lifetime number of starts, wins and earnings, multiple regression models were used, with age, weight, and sex considered as adjusting variables. For the outcomes “number of starts” and “number of wins”, negative binomial regression models were considered for each fitness parameter, since these outcomes are count data and overdispersion is present. Meanwhile, for the outcome “earnings”, gamma regression models were used, as the outcome does not fit a normal distribution, even after log-transformation. Instead, graphical inspection indicates the data approximated the gamma distribution. In order to reduce the risk of type I error due to multiple comparisons, Bonferroni correction was applied, and an adjusted *p* < 0.006 was considered as statistical significance cut-off value for the multiple regression models.

## Results

### Study population

In the present retrospective study, 245 Standardbred racehorses were included, with a median age of 3 (3–4) years and a mean body weight of 453 ± 37 kg, of those 85 females and 160 males (140 stallions and 20 geldings). Mean or median fitness parameters obtained during the incremental treadmill test are displayed in Table 1. In Table 2, the median pre-admission, post-admission, and lifetime racing results are displayed.

**Table 1:** Results of the incremental exercise test on treadmill in the study population.

Variable	Mean ± SD or median (IQR)
V200 (m/s)	7.5 (6.5–8.5)
VLa4 (m/s)	8.0 (6.35–9.2)
Lac max (mmol/L)	22.4 (16.8–27.2)
V max (m/s)	11 (11–11)
pH min	7.1 ± 0.1
Ht max (%)	65.8 ± 4.2

Results are expressed as mean ± standard deviation if normally distributed, or as median (interquartile range) if not normally distributed. V200 = speed at a heart rate of 200 bpm, VLa4 = speed at a plasma lactate concentration of 4 mmol/L, Lac max = peak of plasma lactate concentration, V max = maximum speed, pH min = minimum pH, Ht max = maximum hematocrit.

**Table 2:** Racing results during the 3 months before admission, 6 months after admission, and during the lifetime career.

Variable	3 months before admission	6 months after admission	Lifetime
**Number of starts**	4 (2–5)	4 (2–6)	56.5 (32–96)
**Number of wins**	0 (0–1)	1 (0–2)	9 (5–16)
**Number of placings**	1 (0–2)	2 (1–4)	23 (12–41)
**Earnings (€)**	-	-	63,384 (24,055–127,836)
**Wins/Starts (%)**	0 (0–16.67)	14.29 (0–33.33)	16.03 (10.31–23.16)
**Placings/Starts (%)**	25 (0–50)	50 (25–66.67)	40.34 (29.15–49.39)
**Earnings/Starts (€)**	-	-	939 (504.9–1,840)

Results are expressed as median (interquartile range).

### Fitness parameters *vs* racing results

The significant correlations between fitness parameters and racing results in the whole population and in different age groups during the 3 months before admission, 6 months after discharge, and the lifetime career are displayed, respectively, in Tables 3–5.

**Table 3:** Correlations between fitness parameters and racing results during the 3 months before admission in the whole population and in different age groups. Only significant correlations are displayed.

Fitness parameter	Study population	2-year-old	3-year-old	≥ 4-year-old
**V200**	Placings (r = 0.20, p = 0.003) P:S ratio (r = 0.17, p = 0.012)	ns	Placings (r = 0.27, p = 0.01)	Placings (r = 0.19, p = 0.044) P:S ratio (r = 0.20, p = 0.037)
**VLa4**	Wins (r = 0.14, p = 0.044) Placings (r = 0.23, p = 0.001) P:S ratio (r = 0.20, p = 0.004)	ns	Wins (r = 0.21, p = 0.047) Placings (r = 0.32, p = 0.002) P:S ratio (r = 0.26, p = 0.013)	Placings (r = 0.21, p = 0.024) P:S ratio (r = 0.19, p = 0.04)
**Lac max**	Placings (r = -0.16, p = 0.014)	ns	Placings (r = -0.26, p = 0.014)	ns
**V max**	Placings (r = 0.16, p = 0.017) P:S ratio (r = 0.15, p = 0.028)	ns	Starts (r = 0.28, p = 0.005) Wins (r = 0.23, p = 0.031) Placings (r = 0.30, p = 0.005) W:S ratio (r = 0.21, p = 0.048) P:S ratio (r = 0.27, p = 0.011)	ns
**pH min**	ns	Starts (r = -0.55, p = 0.023)	ns	ns
**Ht max**	Starts (r = 0.36, p<0.001)	Starts (r = 0.63, p = 0.014) Placings (r = 0.71, p = 0.04)	ns	ns

W:S = wins:starts, P:S = placings:starts, E:S = earnings:starts, ns = non-significant.

**Table 4:** Correlations between fitness parameters and racing results during the 6 months after discharge in the whole population and in different age groups. Only significant correlations are displayed.

Fitness parameter	Study population	2-year-old	3-year-old	≥ 4-year-old
**V200**	ns	ns	ns	ns
**VLa4**	Starts (r = 0.16, p = 0.012) Placings (r = 0.17, p = 0.017) P:S ratio (r = 0.15, p = 0.038)	ns	ns	Starts (r = 0.19, p = 0.045) Placings (r = 0.23, p = 0.019) P:S ratio (r = 0.21, p = 0.037)
**Lac max**	ns	ns	ns	ns
**V max**	Starts (r = 0.13, p = 0.049) Placings (r = 0.16, p = 0.019)	P:S ratio (r = 0.52, p = 0.027)	ns	ns
**pH min**	Wins (r = -0.18, p = 0.033) W:S ratio (r = -0.20, p = 0.021)	ns	Wins (r = -0.35, p = 0.007) Placings (r = -0.26, p = 0.047) W:S ratio (r = -0.33, p = 0.011)	ns
**Ht max**	P:S ratio (r = 0.24, p = 0.017)	Wins (r = 0.67, p = 0.039) W:S ratio (r = 0.69, p = 0.034)	ns	ns

W:S = wins:starts, P:S = placings:starts, E:S = earnings:starts, ns = non-significant.

**Table 5:** Correlations between fitness parameters and racing results in the lifetime career in the whole population and in different age groups. Only significant correlations are displayed.

Fitness parameter	Study population	2-year-old	3-year-old	≥ 4-year-old
**V200**	Earnings (r = 0.24, p<0.001) Wins (r = 0.18, p = 0.006, Placings (r = 0.18, p = 0.006) E:S ratio (r = 0.19, p = 0.003) P:S ratio (r = 0.14, p = 0.028)	ns	Starts (r = 0.22, p = 0.032) Earnings (r = 0.34, p = 0.001) Wins (r = 0.27, p = 0.009) Placings (r = 0.29, p = 0.006)	ns
**VLa4**	Starts (r = 0.26, p<0.001) Earnings (r = 0.31, p<0.001) Wins (r = 0.30, p<0.001) Placings (r = 0.31, p<0.001) E:S ratio (r = 0.21, p<0.001) W:S ratio (r = 0.14, p = 0.032) P:S ratio (r = 0.18, p = 0.006)	E:S ratio (r = 0.38, p = 0.049)	Starts (r = 0.26, p = 0.011) Earnings (r = 0.35, p = 0.001) Wins (r = 0.31, p = 0.003) Placings (r = 0.31, p = 0.002)	Starts (r = 0.19, p = 0.043) Earnings (r = 0.21, p = 0.021) Wins (r = 0.29, p = 0.002) Placings (r = 0.26, p = 0.004)
**Lac max**	Earnings (r = -0.16, p = 0.011) Placings (r = -0.15, p = 0.026)	ns	Earnings (r = -0.26, p = 0.013) Wins (r = -0.23, p = 0.029) Placings (r = -0.22, p = 0.035)	ns
**V max**	Starts (r = 0.21, p = 0.001) Earnings (r = 0.35, p<0.001) Wins (r = 0.31,p<0.001) Placings (r = 0.29, p<0.001) E:S ratio (r = 0.33, p<0.001) W:S ratio (r = 0.20, p = 0.002) P:S ratio (r = 0.27, p<0.001)	E:S ratio (r = 0.41, p = 0.03)	ns	Earnings (r = 0.32, p<0.001) Wins (r = 0.31, p = 0.001) Placings (r = 0.26, p = 0.004) E:S ratio (r = 0.30, p = 0.001) W:S ratio (r = 0.23, p = 0.014) P:S ratio (r = 0.23, p = 0.014)
**pH min**	ns	Earnings (r = -0.55, p = 0.024) Wins (r = -0.59, p = 0.014) Placings (r = -0.54, p = 0.027) E:S ratio (r = -0.50, p = 0.045) W:S ratio (r = -0.58, p = 0.017) P:S ratio (r = -0.52, p = 0.036)	ns	ns
**Ht max**	Starts (r = 0.33, p<0.001) Earnings (r = 0.49, p<0.001) Wins (r = 0.39, p<0.001) Placings (r = 0.40, p<0.001) E:S ratio (r = 0.43, p<0.001) W:S ratio (r = 0.29, p = 0.002) P:S ratio (r = 0.34, p<0.001)	Starts (r = 0.72, p = 0.003) Earnings (r = 0.77, p = 0.001) Wins (r = 0.72, p = 0.003) Placings (r = 0.77, p = 0.001) E:S ratio (r = 0.73, p = 0.003) W:S ratio (r = 0.71, p = 0.004) P:S ratio (r = 0.70, p = 0.005)	Earnings (r = 0.32, p = 0.031) Wins (r = 0.32, p = 0.032) W:S ratio (r = 0.34, p = 0.022) P:S ratio (r = 0.34, p = 0.021)	ns

W:S = wins:starts, P:S = placings:starts, E:S = earnings:starts, ns = non-significant.

### Correlations between racing results at different times

The correlations between racing results in the 3 months before admission, 6 months after admission, and in the whole career are displayed in Table 6.

**Table 6:** Correlations between racing results before admission, after discharge, and in the lifetime.

	3 months before admission				
	**Starts**	**Wins**	**Placings**	**W:S ratio**	**P:S ratio**
Lifetime					
**Starts**	*p* < 0.001 r = 0.27	ns	ns	ns	ns
**Wins**	*p* < 0.001 r = 0.27	ns	*p* = 0.045 r = 0.14	ns	ns
**Placings**	*p* < 0.001 r = 0.31	ns	*p* = 0.038 r = 0.14	ns	ns
**Earnings**	*p* < 0.001 r = 0.28	ns	*p* = 0.008 r = 0.18	ns	ns
**W:S ratio**	ns	*p* < 0.0001 r = 0.28	*p* = 0.009 r = 0.18	*p* < 0.001 r = 0.29	*p* = 0.001 r = 0.22
**P:S ratio**	*p* = 0.005 r = 0.18	*p* = 0.001 r = 0.23	*p* < 0.001 r = 0.33	*p* = 0.001 r = 0.23	*p* < 0.001 r = 0.34
**E:S ratio**	*p* = 0.001 r = 0.21	*p* < 0.001 r = 0.20	*p* = 0.001 r = 0.23	*p* = 0.003 r = 0.20	*p* < 0.001 r = 0.24
	6 months after admission				
	**Starts**	**Wins**	**Placings**	**W:S ratio**	**P:S ratio**
Lifetime					
**Starts**	*p* < 0.001 r = 0.38	*p* < 0.001 r = 0.25	*p* < 0.001 r = 0.38	*p* = 0.007 r = 0.19	*p* < 0.001 r = 0.31
**Wins**	*p* < 0.001 r = 0.24	*p* < 0.001 r = 0.44	*p* < 0.001 r = 0.40	*p* < 0.001 r = 0.41	*p* < 0.001 r = 0.41
**Placings**	*p* < 0.001 r = 0.32	*p* < 0.001 r = 0.35	*p* < 0.001 r = 0.45	*p* < 0.001 r = 0.31	*p* < 0.001 r = 0.42
**Earnings**	*p* < 0.001 r = 0.23	*p* < 0.001 r = 0.34	*p* < 0.001 r = 0.30	*p* < 0.001 r = 0.32	*p* < 0.001 r = 0.33
**W:S ratio**	ns	*p* < 0.001 r = 0.39	*p* < 0.001 r = 0.16	*p* < 0.001 r = 0.43	*p* < 0.001 r = 0.25
**P:S ratio**	ns	*p* < 0.001 r = 0.31	*p* < 0.001 r = 0.24	*p* < 0.001 r = 0.34	*p* < 0.001 r = 0.32
**E:S ratio**	ns	*p* < 0.001 r = 0.27	*p* < 0.001 r = 0.15	*p* < 0.001 r = 0.29	*p* = 0.002 r = 0.22

W:S = wins:starts, P:S = placings:starts, E:S = earnings:starts, ns = non significant.

### Predictors of career results

Significant models of negative binomial regression designed for prediction of lifetime number of starts and wins are displayed in Tables 7 and 8, and those of gamma regression for lifetime earnings are shown in Table 9. The full results of all regression analyses are available as supplementary files.

**Table 7:** Multiple negative binomial regression model for lifetime number of starts.

Variable	Coefficient	95% Confidence Interval	*p* value	Significance	
				Classical	Bonferroni
Sex—Mare	ref	ref	ref	ref	ref
Sex—Stallion	0.58	0.40–0.76	< 0.001	***	yes
Sex—Gelding	0.81	0.47–1.15	< 0.001	***	yes
Age	0.11	0.05–0.18	< 0.001	***	yes
Weight	-0.002	-0.004–0.09	0.091	ns	ns
VLa4	0.05	0.01–0.09	0.028	*	ns

Statistical significance is shown as * (p < 0.05), ** (p < 0.01), *** (p < 0.001); ref = reference, ns = non-significant.

**Table 8:** Multiple negative binomial regression models for lifetime number of wins.

Variable	Coefficient	95% Confidence Interval	*p* value	Significance	
				Classical	Bonferroni
Model 1					
Sex—Mare	ref	ref	ref	ref	ref
Sex—Stallion	0.46	0.24–0.68	< 0.001	***	yes
Sex—Gelding	0.50	0.10–0.90	0.015	*	ns
Age	0.18	0.11–0.26	< 0.001	***	yes
Weight	-0.001	-0.004–0.002	0.553	ns	ns
VLa4	0.07	0.02–0.12	0.011	*	ns
Model 2					
Sex—Mare	ref	ref	ref	ref	ref
Sex—Stallion	0.46	0.24–0.68	< 0.001	***	yes
Sex—Gelding	0.52	0.12–0.91	0.011	*	ns
Age	0.15	0.08–0.23	< 0.001	***	yes
Weight	-0.0002	-0.003–0.002	0.835	ns	ns
V max	0.26	0.11–0.42	0.001	***	yes

Statistical significance is shown as * (p < 0.05), ** (p < 0.01), *** (p < 0.001); ref = reference, ns = non-significant.

**Table 9:** Multiple gamma regression models for lifetime earnings.

Variable	Coefficient	95% Confidence Interval	*p* value	Significance	
				Classical	Bonferroni
Model 1					
Sex—Mare	ref	ref	ref	ref	ref
Sex—Stallion	0.18	-0.18–0.54	0.328	ns	ns
Sex—Gelding	-0.42	-1.08–0.25	0.221	ns	ns
Age	0.30	0.17–0.43	< 0.001	***	yes
Weight	-0.002	-0.01–0.004	0.924	ns	ns
V200	0.21	0.08–0.34	0.002	**	yes
Model 2					
Sex—Mare	ref	ref	ref	ref	ref
Sex—Stallion	0.20	-0.18–0.59	0.304	ns	ns
Sex—Gelding	-0.41	-1.13–0.30	0.255	ns	ns
Age	0.31	0.17–0.46	< 0.001	***	yes
Weight	-0.0002	-0.01–0.004	0.926	ns	ns
VLa4	0.10	0.02–0.18	0.017	*	ns
Model 3					
Sex—Mare	ref	ref	ref	ref	ref
Sex—Stallion	0.26	-0.10–0.62	0.153	ns	ns
Sex—Gelding	-0.34	-1.02–0.34	0.328	ns	ns
Age	0.23	0.09–0.37	0.001	***	yes
Weight	0.001	-0.004–0.01	0.611	ns	ns
V max	0.49	0.24–0.75	< 0.001	***	yes

Statistical significance is shown as * (p < 0.05), ** (p < 0.01), *** (p < 0.001); ref = reference, ns = non-significant.

## Discussion

Among the different fitness parameters investigated in the present study, V200 and VLa4 seem to be the most useful for the evaluation of poor performance in ≥ 3-year-old Standardbred racehorses, as they are related with the racing results in the period just prior to admission; also, V max and Lac max deserve to be considered exclusively in 3-year-old horses. In the whole population, the total number of starts in career is associated with VLa4, the total number of wins with VLa4 and V max, while total earnings with VLa4, V200, and V max. In particular, VLa4 may be useful for career prediction in adult horses, while Ht max and pH min seem good predictors of lifetime performance in 2-year-old horses only. However, the strength of these relationships is sometimes just weak or moderate: therefore, fitness parameters obtained during treadmill test should be considered cautiously in poorly-performing Standardbred trotter horses.

Exercise tests are recognized as a good tool for the assessment of the fitness status in sport horses [5–7, 28–32]; however, the associations between different fitness parameters and racing results have been questioned [1, 5, 12–17]. Various explanations have been proposed for the discrepancies in previous reports. First of all, the type of exercise test may significantly influence the meaning of the results; for example, some authors only used tests on the field [1, 13–16], which allow the evaluation of the horses in the environment where they normally get trained, while others performed tests on the treadmill [5, 12, 17], which have the advantage of being more controlled and standardized. In fact, the type of ground surface, the weather conditions and the driver can influence the tested parameters, while exercise on treadmill is highly repeatable as the ground surface is always the same, and the speed, inclination, temperature, and aeration can be controlled. Therefore, the treadmill test reduces the external biases and can provide an objective assessment of the fitness in racehorses. Another reason for disagreements in previous studies evaluating the relationships between fitness parameters and treadmill is the lack of standardization for racing performance evaluation: for example, the number of starts in the lifetime is a good measure for athletic longevity but does not necessarily correspond to a good athletic quality; conversely, wins and earnings mainly reflect racing success. However, also in the case of earnings, the quality is not ensured: in fact, earnings are considered important for the horse pricing at sales, but there are many biases which could interfere with this variable, such as racing class, sex, country, etc.; the parameter “earnings per start” may be better but is reported to have a minor statistical power. Similarly, the numbers of wins and placings are influenced by the racing class [19]. For this reason, in the present study, we chose to evaluate a combination of different previously reported parameters, and the ratios between each parameter and the number of starts; in this way, we aimed to obtain results which should better reflect racing successfulness. Furthermore, we decided to evaluate racing results in the period immediately before and after the treadmill test, besides in the whole lifetime; in fact, only poorly performing horses were included in our study, and so the test was performed when horses were not at their best condition. Therefore, the predictive value of our test for the whole career could have been questionable.

Another main point is that results of previous studies can be difficult to compare, as different fitness parameters were investigated; in general, blood lactate concentration and heart rate are considered as the main indices for fitness monitoring, but they can be assessed in different moments and related with other measures, resulting in different parameters. For example, V200 and VLa4 indicate the heart rate and the blood lactate concentration in relation to speed; other adopted parameters are the maximum heart rate and lactate concentration reached during the test, while their post-exercise assessment can reflect recovery. In our study, the VLa4 was the parameter correlated with the greatest number of racing results variables. In particular, it was positively correlated with wins and placings before admission, reflecting the poor performance condition for which horses were referred for examination, in ≥ 3-year-old horses. Conversely, in 2-year-old horses, VLa4 was not associated with racing results during the poor performance period. Moreover, in adult horses (≥ 4-year-old), VLa4 was positively correlated with the number of starts and placings in the 6 months after examination, indicating racing potential after recovery. Finally, only in ≥ 3-year-old horses, it was correlated with all the career-related parameters, including results related to longevity and quality. These findings suggest that horses whose fitness status was minimally affected remained the best in the population, considering both the short- and long-term performance outcomes. Likewise, horses that were evaluated only after their fitness was very impaired tended to maintain poorer performance after examination and had a less successful lifetime career. Moreover, the absence of correlations in 2-year-olds suggests that, at the moment of treadmill examination, younger horses had not reached yet the fitness that would represent their career: therefore, we suggest that studies on performance should consider 2-year-old horses separately for older horses. In contrast, a previous study reported a stronger correlation between VLa4 and the index of trot in the year of examination in younger horses, hypothesizing that the relative role of aerobic capacity in performance may decrease with age, when performance-limiting factors such as musculoskeletal or other subclinical conditions may increase [13]. In the multivariate models, considering age, sex, and weight as confounding factors, VLa4 was the only parameter which remained significant for the outcomes related to the total number of starts, wins, and earnings in the study population. For this reason, VLa4 is confirmed to be the most important physiological parameter that should be measured for performance evaluation and prediction in Standardbred racehorses. Similar results supporting the significance of VLa4 in racing performance profiling have been reported by several studies [5, 12–14]. Indeed, VLa4 is considered a good predictor of aerobic capacity of horses, essential during maximal racing exercise, and an indication of a superior exercise capacity [7, 13, 17].

Other research groups investigated the associations between performance and post-exercise blood lactate concentration. In exercise tests, the peak of lactate, usually reached within 5 minutes post-exercise, is thought to be associated with the anaerobic capacity of horses, but no strong evidence has been obtained yet [33]. In the present study, the peak of lactate was inversely correlated with the number of placings in the 3 months before examination, and with the lifetime number of wins, placings and earnings, exclusively in 3-year-old horses. However, the correlation coefficients were weak and the lack of significance in 2-year-olds and in adult horses suggests that this parameter should be interpreted cautiously. In fact, its clinical meaning is not clear: indeed, it is thought to reflect a good anaerobic capacity, but may also be a consequence of prolonged lactate accumulation due to a low aerobic threshold. This may explain why previous study report either a positive [1, 34], negative [17], or absent [16, 35] association with racing performance.

Among the heart rate-related parameters, V200 is of special interest, as it is easily obtainable and has been associated with better racing results by some [5, 13], but not all [14]. studies. In our study, only in ≥ 3-year-old horses, V200 was positively but weakly correlated with placings in the period before admission, and with all career results (longevity and quality). In multivariate models, V200 was only predictive of total career earnings, but not of the number of starts and wins. Our results confirm the usefulness of V200 as an index for fitness evaluation when investigating poor performance, but VLa4 should be preferred, as more strongly correlated to racing outcomes. This may be due to the fact that the maximum heart rate is variable between subjects: therefore, different individuals may be exercising at a different proportion of their maximum heart rate at a fixed heart rate value, such as 200 bpm [33]. Consequently, this variable is less useful to predict maximal aerobic capacity.

In the present study, we also took into consideration the maximum speed reached during the incremental test before the onset of fatigue. It was not a surprise that it was positively correlated with all racing outcomes before admission in 3-year-old horses, and in the whole career in adult horses. Moreover, it was significant in multivariate models for the prediction of total number of wins and earnings. Therefore, our results suggest that V max should be considered as one of the most important parameters for poor performance evaluation and career success prediction.

While most previous studies limited investigation to few fitness parameters, in the present study we also decided to include the maximum hematocrit and the minimum blood pH reached during the test. A positive correlation between hematocrit after splenic contraction and racing times in trotters was first reported in the seventies [18], while no correlation between post-exercise hematocrit and handicap ratings was detected in Thoroughbreds [17]. Well-performing Standardbreds showed higher resting and post-exercise hematocrit values compared to poorly performing in another study [5]. In 2-year-old horses, our results showed a positive strong correlation between maximum hematocrit and number of starts and placements in the period prior hospitalization, and with the number of wins and wins:starts ratio in the following period. Similarly, in 2- and 3-year-olds, Ht max was positively correlated with all the lifetime parameters, suggesting that a higher hematocrit is associated with a longer and successful career in young horses, but not in adults. Moreover, the correlation coefficient was higher in 2-year-olds, suggesting a decrease of clinical meaning of this parameter with increasing age and training level. Furthermore, in multivariate models considering age as confounding factor, the Ht max was not predictive of lifetime career parameters. We hypothesize that the hematocrit may be of special interest exclusively in young horses as it may reflect their potential in oxygen-carrying capacity, while in adult horses it may lose importance as the aerobic capacity can be trained and many more biases may interfere with performance. Analogously, the minimum pH reached during the test was inversely correlated with the number of wins and placings in the 6 months after examination, and with all the lifetime results, but only in 2-year-old horses. The minimum pH reached during the test may represent an index of the organism buffer capacity or may reflect lactic acid accumulation. However, the reasons behind these results are unknown and further investigation is needed to clarify their clinical meaning.

Although many significant correlations have been observed, it must be highlighted that, in the present study, the correlation coefficients were overall weak, probably because many other factors contribute to racing outcomes, besides a horse’s fitness status. First, biomechanical and locomotory parameters can be very important in the prediction of racing results, including stride length, frequency, and symmetry. Genetics also plays of a central role in determining performance, together with training and racing strategies. However, in cohort retrospective studies based on convenience samples it is very difficult to consider and control all these variables, which represents a main limitation for this kind of study.

Another main limitation of the present study consists of the inclusion of only poorly performing horses; therefore, some possible relationships between fitness parameters and racing performance may have been missed, as no data on good performers were obtained and the range of performance quality was reduced. However, our study was conducted retrospectively on clinical patients referred to our hospital for the investigation of poor performance. In the future, it may be interesting to compare fitness parameters also of fit horses to identify any further associations or predictive variables. Therefore, this study’s results should be considered valid only for poorly performing Standardbred racehorses. From a statistical point of view, another limitation of the present study is represented by the high number of tests performed, which may have over-identified statistical associations; therefore, to reduce the risk of type I error, we included Bonferroni correction for multiple comparisons.

Concerning the predictive value of the racing results in the period around examination, significant correlations with lifetime career were identified for both the 3-month period before admission, and the following 6 months. First, horses taking part in more races in the 3 months before admission had a longer and more successful career. Similarly, the number of wins and placings in the 3 months before admission were correlated to a better quality of the lifetime career. Also the quality, expressed as the ratios between wins and starts and placings and starts, before admission was correlated with the lifetime quality, including average earnings per start. The racing results in the 6 months after examination were more strongly correlated with the whole career, probably because they better reflect the horse quality, as it has presumably been treated for poor performance and has returned to good fitness. Horses taking part in more races after examination also had a longer and more successful career. Moreover, the number of wins, placings, and their ratios in relation to the number of starts, were positively correlated with all the lifetime outcome parameters, suggesting that they are good indices of longevity and quality of the career. These results suggest that monitoring race outcomes in the 6 months after examination may be useful for the prediction of the lifetime results, and for supporting trainers to take decisions on their horses’ careers.

## Conclusions

In conclusion, the present study confirms the usefulness of standardized treadmill test for the evaluation of poor performance and the prediction of racing outcomes in Standardbred racehorses. In particular, VLa4 and maximum speed seem to be the most significant parameters in adult horses, while the maximum hematocrit and the minimum pH reached during the test may represent interesting indices of performance potential in 2-year-old horses. Moreover, the monitoring of racing results in the period right after hospitalization may aid to the decision-making process on Standardbreds’ career.

## Supporting information

S1 File (PDF)

S1 Dataset (XLSX)
