# Supplementary material for: Evaluation of fitness parameters in relation to racing results in 245 Standardbred trotter horses submitted for poor performance examination: A retrospective study
Source: PLoS One. 2023 Oct 19;18(10):e0293202. doi: 10.1371/journal.pone.0293202 (PMC10586626; doi:10.1371/journal.pone.0293202)
Supplement: S1 File — (PDF) [file pone.0293202.s001.pdf]

Lifetime number of starts

Negative binomial regression — Number of obs = 237; LR chi2(5) = 66.89; Prob > chi2 = 0.0000; Pseudo R2 = 0.0275

Dispersion: mean

Log likelihood = -1182.8048

CORSEINCARRIERA	Coefficient	Std. err.	z	P>|z|	[95% conf.	interval]
sesso						
Stallion	.5838427	.0934084	6.25	0.000	.4007655	.7669198
Gelding	.8338386	.1738283	4.80	0.000	.4931415	1.174536
AGE	.1175716	.0320929	3.66	0.000	.0546706	.1804726
WEIGHT	-.0020609	.00122	-1.69	0.091	-.004452	.0003303
V200	.0270206	.0355798	0.76	0.448	-.0427145	.0967557
_cons	4.039096	.5954116	6.78	0.000	2.87211	5.206081
/lnalpha	-.9118643	.0927642			-1.093679	-.7300497
alpha	.4017745	.0372703			.3349819	.481885

LR test of alpha=0: chibar2(01) = 4440.48 Prob >= chibar2 = 0.000

Negative binomial regression — Number of obs = 237; LR chi2(5) = 71.02; Prob > chi2 = 0.0000; Pseudo R2 = 0.0292

Dispersion: mean

Log likelihood = -1180.7375

CORSEINCARRIERA	Coefficient	Std. err.	z	P>|z|	[95% conf.	interval]
sesso						
Stallion	.5814397	.0926674	6.27	0.000	.3998149	.7630645
Gelding	.811602	.1724566	4.71	0.000	.4735933	1.149611
AGE	.1128579	.0319228	3.54	0.000	.0502903	.1754255
WEIGHT	-.0020403	.0012088	-1.69	0.091	-.0044095	.000329
VLA4	.0476756	.0216455	2.20	0.028	.0052513	.0900999
_cons	3.880483	.5558911	6.98	0.000	2.790957	4.97001
/lnalpha	-.9288391	.0929816			-1.11108	-.7465985
alpha	.395012	.0367289			.3292033	.4739761

LR test of alpha=0: chibar2(01) = 4340.68 Prob >= chibar2 = 0.000

```
Negative binomial regression                          Number of obs =    237
                                                      LR chi2(5)    =  66.45
Dispersion: mean                                      Prob > chi2   = 0.0000
Log likelihood = -1183.0202                           Pseudo R2     = 0.0273
```

CORSEINCARRIERA	Coefficient	Std. err.	z	P>|z|	[95% conf.	interval]
sesso						
Stallion	.5860977	.0934008	6.28	0.000	.4030356	.7691599
Gelding	.8434447	.1733208	4.87	0.000	.5037422	1.183147
AGE	.1184268	.0322649	3.67	0.000	.0551888	.1816648
WEIGHT	-.0020654	.0012198	-1.69	0.090	-.0044562	.0003253
LACMAX	-.0019137	.0050091	-0.38	0.702	-.0117313	.007904
_cons	4.280728	.5515641	7.76	0.000	3.199682	5.361774
/lnalpha	-.9101529	.0927472			-1.091934	-.7283718
alpha	.4024627	.0373273			.3355669	.4826943

LR test of alpha=0: chibar2(01) = 4444.00   Prob >= chibar2 = 0.000

```
Negative binomial regression                          Number of obs =    237
                                                      LR chi2(5)    =  68.35
Dispersion: mean                                      Prob > chi2   = 0.0000
Log likelihood = -1182.0738                           Pseudo R2     = 0.0281
```

CORSEINCARRIERA	Coefficient	Std. err.	z	P>|z|	[95% conf.	interval]
sesso						
Stallion	.5853969	.0931134	6.29	0.000	.4028981	.7678957
Gelding	.8308184	.1730963	4.80	0.000	.4915559	1.170081
AGE	.1036001	.0334939	3.09	0.002	.0379532	.169247
WEIGHT	-.001797	.0012291	-1.46	0.144	-.0042061	.000612
VMAX	.0960719	.0670518	1.43	0.152	-.0353473	.227491
_cons	3.117025	.9439154	3.30	0.001	1.266985	4.967065
/lnalpha	-.9180178	.0928569			-1.100014	-.7360217
alpha	.3993098	.0370786			.3328665	.4790158

LR test of alpha=0: chibar2(01) = 4387.45   Prob >= chibar2 = 0.000

Negative binomial regression — Number of obs = 157; LR chi2(5) = 53.92; Prob > chi2 = 0.0000; Pseudo R2 = 0.0338

Dispersion: mean
Log likelihood = -771.00335

CORSEINCARRIERA	Coefficient	Std. err.	z	P>|z|	[95% conf.	interval]
sesso						
Stallion	.5426315	.1124415	4.83	0.000	.3222503	.7630127
Gelding	.8388048	.2010985	4.17	0.000	.444659	1.232951
AGE	.1669153	.0401999	4.15	0.000	.088125	.2457057
WEIGHT	-.0020257	.0014075	-1.44	0.150	-.0047843	.000733
pHMIN	-.6763817	.5559273	-1.22	0.224	-1.765979	.4132157
_cons	8.827867	3.976535	2.22	0.026	1.034001	16.62173
/lnalpha	-.9503376	.1155357			-1.176783	-.7238919
alpha	.3866105	.0446673			.3082687	.4848616

LR test of alpha=0: chibar2(01) = 2582.59 Prob >= chibar2 = 0.000

Negative binomial regression — Number of obs = 118; LR chi2(5) = 43.73; Prob > chi2 = 0.0000; Pseudo R2 = 0.0369

Dispersion: mean
Log likelihood = -571.02827

CORSEINCARRIERA	Coefficient	Std. err.	z	P>|z|	[95% conf.	interval]
sesso						
Stallion	.5072347	.1336211	3.80	0.000	.2453423	.7691272
Gelding	.7146465	.2453737	2.91	0.004	.2337228	1.19557
AGE	.1583125	.0467527	3.39	0.001	.0666789	.2499462
WEIGHT	-.0013425	.0017177	-0.78	0.434	-.0047091	.002024
HtMAX	.0165502	.0171696	0.96	0.335	-.0171015	.0502019
_cons	2.600214	1.166401	2.23	0.026	.3141102	4.886317
/lnalpha	-.9637398	.1348984			-1.228136	-.6993438
alpha	.3814636	.0514588			.292838	.4969113

LR test of alpha=0: chibar2(01) = 1721.82 Prob >= chibar2 = 0.000

Lifetime number of wins

```
Negative binomial regression                            Number of obs =    236
                                                        LR chi2(5)    =  55.83
Dispersion: mean                                        Prob > chi2   = 0.0000
Log likelihood = -784.9277                              Pseudo R2     = 0.0343
```

VITTORIE	Coefficient	Std. err.	z	P>|z|	[95% conf.	interval]
sesso						
Stallion	.4591648	.1143879	4.01	0.000	.2349686	.6833611
Gelding	.5202215	.207529	2.51	0.012	.1134721	.9269708
AGE	.1917941	.0385965	4.97	0.000	.1161462	.2674419
WEIGHT	-.0010371	.0014333	-0.72	0.469	-.0038464	.0017722
V200	.0670979	.0429842	1.56	0.119	-.0171496	.1513453
_cons	1.283466	.6983917	1.84	0.066	-.0853569	2.652288
/lnalpha	-.6870062	.1193879			-.9210023	-.4530101
alpha	.5030799	.0600617			.3981198	.6357117

```
LR test of alpha=0: chibar2(01) = 598.07                Prob >= chibar2 = 0.000
```

Negative binomial regression

Number of obs = 236
LR chi2(5) = 53.82
Prob > chi2 = 0.0000
Pseudo R2 = 0.0331

Dispersion: mean
Log likelihood = -785.93246

VITTORIE	Coefficient	Std. err.	z	P>|z|	[95% conf.	interval]
sesso						
Stallion	.4875638	.1140391	4.28	0.000	.2640513	.7110762
Gelding	.5457833	.2080831	2.62	0.009	.137948	.9536186
AGE	.197719	.0390992	5.06	0.000	.121086	.274352
WEIGHT	-.0010489	.0014344	-0.73	0.465	-.0038604	.0017625
LACMAX	.0037974	.0059752	0.64	0.525	-.0079137	.0155085
_cons	1.665945	.6445701	2.58	0.010	.4026104	2.929279
/lnalpha	-.674367	.1188109			-.9072321	-.441502
alpha	.5094788	.0605316			.4036399	.6430698

LR test of alpha=0: chibar2(01) = 613.37 Prob >= chibar2 = 0.000

Negative binomial regression

Number of obs = 236
LR chi2(5) = 64.13
Prob > chi2 = 0.0000
Pseudo R2 = 0.0394

Dispersion: mean
Log likelihood = -780.77681

VITTORIE	Coefficient	Std. err.	z	P>|z|	[95% conf.	interval]
sesso						
Stallion	.4603236	.1114771	4.13	0.000	.2418326	.6788147
Gelding	.5156633	.2030916	2.54	0.011	.117611	.9137156
AGE	.1530106	.0392999	3.89	0.000	.0759842	.2300369
WEIGHT	-.0002966	.001422	-0.21	0.835	-.0030837	.0024905
VMAX	.2636097	.0793039	3.32	0.001	.1081768	.4190425
_cons	-1.310198	1.10677	-1.18	0.236	-3.479427	.8590305
/lnalpha	-.7363353	.1214024			-.9742795	-.498391
alpha	.4788656	.0581354			.3774642	.6075073

LR test of alpha=0: chibar2(01) = 547.56 Prob >= chibar2 = 0.000

```
Negative binomial regression                            Number of obs =    156
                                                        LR chi2(5)    =  42.34
Dispersion: mean                                        Prob > chi2   = 0.0000
Log likelihood = -506.25913                             Pseudo R2     = 0.0401
```

VITTORIE	Coefficient	Std. err.	z	P>|z|	[95% conf.	interval]
sesso						
Stallion	.4245755	.1372569	3.09	0.002	.1555568	.6935941
Gelding	.4542049	.2411843	1.88	0.060	-.0185078	.9269175
AGE	.2469724	.0490774	5.03	0.000	.1507825	.3431623
WEIGHT	-.0001153	.0016705	-0.07	0.945	-.0033894	.0031588
pHMIN	-.9940952	.6727922	-1.48	0.140	-2.312744	.3245532
_cons	8.217215	4.788814	1.72	0.086	-1.168688	17.60312
/lnalpha	-.7353862	.1512531			-1.031837	-.4389357
alpha	.4793203	.0724987			.3563518	.6447223

```
LR test of alpha=0: chibar2(01) = 340.29               Prob >= chibar2 = 0.000
```

```
Negative binomial regression                            Number of obs =    117
                                                        LR chi2(5)    =  30.77
Dispersion: mean                                        Prob > chi2   = 0.0000
Log likelihood = -380.75833                             Pseudo R2     = 0.0388
```

VITTORIE	Coefficient	Std. err.	z	P>|z|	[95% conf.	interval]
sesso						
Stallion	.3550883	.1743095	2.04	0.042	.013448	.6967286
Gelding	.4942589	.3202415	1.54	0.123	-.1334029	1.121921
AGE	.1801149	.0612966	2.94	0.003	.0599758	.3002541
WEIGHT	.0004108	.0022397	0.18	0.854	-.0039789	.0048005
HtMAX	.0435791	.0237393	1.84	0.066	-.0029492	.0901073
_cons	-1.723851	1.595482	-1.08	0.280	-4.850938	1.403235
/lnalpha	-.5616262	.1695674			-.8939722	-.2292802
alpha	.5702809	.0967011			.4090278	.7951057

```
LR test of alpha=0: chibar2(01) = 311.38               Prob >= chibar2 = 0.000
```

Lifetime earnings

```
Iteration 0:   log likelihood = -2960.1421
Iteration 1:   log likelihood = -2950.9388
Iteration 2:   log likelihood = -2950.9221
Iteration 3:   log likelihood = -2950.9221

Generalized linear models                         Number of obs   =        237
Optimization     : ML                             Residual df     =        231
                                                  Scale parameter =   1.583297
Deviance         =  558.4569243                   (1/df) Deviance =   2.417562
Pearson          =  365.7415648                   (1/df) Pearson  =   1.583297

Variance function: V(u) = u^2                     [Gamma]
Link function    : g(u) = ln(u)                   [Log]

                                                  AIC             =   24.95293
Log likelihood   = -2950.922052                   BIC             =   -704.665
```

EARNINGSCARRIERA	Coefficient	OIM std. err.	z	P>|z|	[95% conf.	interval]
sesso						
Stallion	.180701	.1846852	0.98	0.328	-.1812753	.5426773
Gelding	-.4167319	.340166	-1.23	0.221	-1.083445	.2499811
AGE	.3008478	.0664593	4.53	0.000	.17059	.4311056
WEIGHT	-.0002399	.0025056	-0.10	0.924	-.0051507	.004671
V200	.210262	.0678074	3.10	0.002	.0773619	.3431621
_cons	8.775486	1.216979	7.21	0.000	6.390251	11.16072

```
Iteration 0:   log likelihood = -2963.0571
Iteration 1:   log likelihood = -2953.6276
Iteration 2:   log likelihood = -2953.6171
Iteration 3:   log likelihood = -2953.6171

Generalized linear models                         Number of obs   =        237
Optimization     : ML                             Residual df     =        231
                                                  Scale parameter =   1.817534
Deviance         =  563.8470216                   (1/df) Deviance =   2.440896
Pearson          =  419.8503295                   (1/df) Pearson  =   1.817534

Variance function: V(u) = u^2                     [Gamma]
Link function    : g(u) = ln(u)                   [Log]

                                                  AIC             =   24.97567
Log likelihood   =   -2953.6171                   BIC             =  -699.2749
```

EARNINGSCARRIERA	Coefficient	OIM std. err.	z	P>|z|	[95% conf.	interval]
sesso						
Stallion	.2035479	.1981772	1.03	0.304	-.1848722	.5919681
Gelding	-.4144786	.3642289	-1.14	0.255	-1.128354	.299397
AGE	.3127914	.0731876	4.27	0.000	.1693463	.4562365
WEIGHT	-.0002461	.0026643	-0.09	0.926	-.005468	.0049758
VLA4	.1002525	.0420278	2.39	0.017	.0178795	.1826256
_cons	9.524221	1.215644	7.83	0.000	7.141602	11.90684

```
Iteration 0:   log likelihood = -2969.1295
Iteration 1:   log likelihood = -2958.2937
Iteration 2:   log likelihood = -2958.2795
Iteration 3:   log likelihood = -2958.2795

Generalized linear models                         Number of obs   =        237
Optimization     : ML                             Residual df     =        231
                                                  Scale parameter =   1.928412
Deviance         =  573.1718054                   (1/df) Deviance =   2.481263
Pearson          =  445.4632134                   (1/df) Pearson  =   1.928412

Variance function: V(u) = u^2                     [Gamma]
Link function    : g(u) = ln(u)                   [Log]

                                                  AIC             =   25.01502
Log likelihood   = -2958.279492                   BIC             =  -689.9501
```

EARNINGSCARRIERA	Coefficient	OIM std. err.	z	P>|z|	[95% conf.	interval]
sesso						
Stallion	.2829579	.2024008	1.40	0.162	-.1137403	.6796561
Gelding	-.3180844	.3728435	-0.85	0.394	-1.048844	.4126754
AGE	.3147241	.0759897	4.14	0.000	.165787	.4636613
WEIGHT	-.0009332	.0027095	-0.34	0.731	-.0062437	.0043773
LACMAX	-.0045436	.0103341	-0.44	0.660	-.0247981	.0157108
_cons	10.67116	1.188426	8.98	0.000	8.341891	13.00043

```
Iteration 0:   log likelihood = -2955.2302
Iteration 1:   log likelihood = -2946.5813
Iteration 2:   log likelihood = -2946.5594
Iteration 3:   log likelihood = -2946.5594

Generalized linear models                         Number of obs   =        237
Optimization     : ML                             Residual df     =        231
                                                  Scale parameter =   1.649879
Deviance         =  549.7315665                   (1/df) Deviance =    2.37979
Pearson          =  381.1219357                   (1/df) Pearson  =   1.649879

Variance function: V(u) = u^2                     [Gamma]
Link function    : g(u) = ln(u)                   [Log]

                                                  AIC             =   24.91611
Log likelihood   = -2946.559373                   BIC             =  -713.3903

------------------------------------------------------------------------------------
                 |                 OIM
EARNINGSCARRIERA | Coefficient  std. err.      z    P>|z|     [95% conf. interval]
-----------------+------------------------------------------------------------------
           sesso |
       Stallion  |   .2636103    .184302     1.43   0.153    -.0976149    .6248355
        Gelding  |  -.3385667   .3463283    -0.98   0.328    -1.017358    .3402244
                 |
             AGE |   .2289528   .0703656     3.25   0.001     .0910387    .3668668
          WEIGHT |    .001319   .0025911     0.51   0.611    -.0037594    .0063974
            VMAX |   .4935069   .1286875     3.83   0.000      .241284    .7457298
           _cons |   4.417938    1.92327     2.30   0.022     .6483979    8.187477
------------------------------------------------------------------------------------
```

```
Iteration 0:   log likelihood = -1960.0617
Iteration 1:   log likelihood =  -1951.711
Iteration 2:   log likelihood = -1951.6943
Iteration 3:   log likelihood = -1951.6943

Generalized linear models                         Number of obs   =        157
Optimization     : ML                             Residual df     =        151
                                                  Scale parameter =   2.509438
Deviance         =  425.2165926                   (1/df) Deviance =   2.816004
Pearson          =  378.9252076                   (1/df) Pearson  =   2.509438

Variance function: V(u) = u^2                     [Gamma]
Link function    : g(u) = ln(u)                   [Log]

                                                  AIC             =   24.93878
Log likelihood   = -1951.694332                   BIC             =  -338.2765
```

EARNINGSCARRIERA	Coefficient	OIM std. err.	z	P>|z|	[95% conf.	interval]
sesso						
Stallion	.2173875	.2888992	0.75	0.452	-.3488446	.7836197
Gelding	-.3503601	.5057575	-0.69	0.488	-1.341627	.6409065
AGE	.3347025	.1076409	3.11	0.002	.1237301	.5456748
WEIGHT	-.001516	.0037334	-0.41	0.685	-.0088333	.0058012
pHMIN	.0841951	1.451002	0.06	0.954	-2.759717	2.928107
_cons	10.15559	10.33506	0.98	0.326	-10.10074	30.41193

```
Iteration 0:   log likelihood = -1455.9439
Iteration 1:   log likelihood =  -1449.733
Iteration 2:   log likelihood = -1449.6912
Iteration 3:   log likelihood = -1449.6912

Generalized linear models                          Number of obs   =        118
Optimization     : ML                              Residual df     =        112
                                                   Scale parameter =   2.539734
Deviance         =  358.9041914                    (1/df) Deviance =   3.204502
Pearson          =  284.4501576                    (1/df) Pearson  =   2.539734

Variance function: V(u) = u^2                      [Gamma]
Link function    : g(u) = ln(u)                    [Log]

                                                   AIC             =   24.67273
Log likelihood   = -1449.691211                    BIC             =  -175.4125
```

EARNINGSCARRIERA	Coefficient	OIM std. err.	z	P>|z|	[95% conf.	interval]
sesso						
Stallion	.0607407	.3592361	0.17	0.866	-.6433492	.7648306
Gelding	-.3643962	.6456131	-0.56	0.572	-1.629775	.9009821
AGE	.3393387	.1300672	2.61	0.009	.0844116	.5942658
WEIGHT	-.0042341	.0048312	-0.88	0.381	-.0137031	.0052349
HtMAX	.0747981	.0508098	1.47	0.141	-.0247871	.1743834
_cons	6.984877	3.034922	2.30	0.021	1.036539	12.93322
